# Steel Uniformity

**Published:** 1885-02

**Authors:** 


### Steel Uniformity.

The users of steel for manufacturing purposes, and probably the producers of steel, would welcome any information that would insure uniformity in the product. It appears to be almost a waste of investigating endeavor to argue on the relative merits of steel produced from the iron and that cemented from the bar. The true test of their relative merits is that of use in practice. Yet there seems to be an almost insane desire to turn all our iron into steel, and to produce steel as directly from the ore as pig iron is produced. An enthusiast recently called attention to some lathe and planing tools, cast from iron melted in the cupola in the regular way, and then submitted to a cementing process of brief duration, claiming them to be true cast steel, or its equivalent. And there are others who assume that all the work of cementation and the after processes may be dispensed with, and good tool steel result.

This nonsense will be taken up and repeated by mechanics who may be like the Athenians described in Acts xvii., 21 ; but there are workers who know steel from carbonized cast iron, and who require for their work all the proper qualities of cast steel.

What is needed in regard to steel information is how to make cast steel to-day, to-morrow, and so on indefinitely, the same. We know that iron can be refined, and that its components can be changed, so as to improve its quality, and so that it can assume some of the qualities of cast steel, and be called steel commercially. But what is required is an equable quality of the steel used for tools.

This equable quality does not exist among the steels made by the best known manufacturers; they may claim it, but the facts of practice do not sustain the claim. All the differences in working different lots from the same makers, in working different bars from the same lot, in working from the same bar, do not come from the difference in treatment and manipulation. A chart of tests comprehending the steels of five of the best known manufacturers of steels show not only a difference between the products of the different establishments, but a great lack of uniformity in the specimens tested from the same maker. An establishment that makes the production of small steel tools a specialty; and is probably as successful as any other in this country, or other countries, has its tools returned for failure in exactly opposite directions—too soft, too brittle. What is to be done? There is the same treatment of, commercially, the same material. The fact is that uniformity in the character of crucible steel is an attainment yet to be reached, and it is time that scientific and practical men devoted their attention to this attainment, instead of arguing on the identity of purified iron, called "Bessemer steel," and cast steel *per se.*

---

To cure an abscess without a cicatrix, Dr. Quinlan uses a silver wire passed through the abscess, before it has reached the skin, and retained there. It acts as a drain, he says, and has never failed in his hands.—*Med. and Surg. Reporter.*

